# Reservoir damage mechanism and “Double protection” drilling fluid of tight sandstone gas reservoir in central SiChuan Basin

**DOI:** 10.1038/s41598-024-62696-7

**Published:** 2024-05-31

**Authors:** Yu Fan, Weian Huang, Jianhua Guo, Ruiyv Bai, Lin Jiang, Zengbao Wang

**Affiliations:** 1 School of Petroleum Engineering, China University of Petroleum (East China), Qingda, 266580 Shandong People’s Republic of China; 2 Engineering Technology Research Institute of CNPC Southwest Oil and Gas Field Branch, Chengdu, 610017 Sichuan People’s Republic of China; 3 National Key Laboratory of Deep Oil and Gas, China University of Petroleum (East China), Qingdao, 266580 People’s Republic of China

**Keywords:** Tight sandstone gas reservoir, Sensitivity damage, Reservoir protection, Environmental protection, Drilling fluid, Chemistry, Environmental chemistry

## Abstract

It is very important to clarify the reservoir damage characteristics and damage characteristics and damage mechanism of tight sandstone gas reservoirs in Jinhua-Zhongtai mountain area of central SiChuan Basin, and put forward the technical countermeasures of “Double protection” drilling fluid to protect the reservoir and the environment, which is very important for the efficient well construction in this area. Through X-ray diffraction, scanning electron microscopy, casting thin sections and other testing methods, the mineral composition and microstructure characteristics of the block were analyzed, and the potential damage factors of the reservoir were clarified. Based on the sensitivity evaluation, it was revealed that the overall sensitivity damage of the block was weak. The main damage type was salt-sensitive damage, and the critical salinity was 9472.5 mg/L. On the basis of the environmental protection drilling fluid system used in this block, the surfactant which can effectively prevent gas invasion and reduce surface tension is selected, and the “Double protection” drilling fluid system is constructed. Through comprehensive performance test and reservoir protection performance evaluation, the core permeability damage rate of the optimized drilling fluid system is reduced from 88.77 to 18.66%, and the cuttings recovery rate is increased to more than 66%, and the cuttings expansion rate is reduced to less than 3.2%, which can effectively solve the problem of reservoir damage in drilling in Jinhua-Zhongtai mountain block in central Sichuan.

## Introduction

China is rich in tight sandstone gas reserves, with a total geological resource of 20.91012 m^3^. It is widely distributed in the four basins of Ordos, Sichuan, Tarim and Songliao, and has great mining potential^1^. The formation of tight sandstone reservoirs is mainly due to the decrease of reservoir porosity and permeability caused by diagenesis. In addition, during the conversion of organic matter into oil and gas in the formation, carbonate rocks and some skeleton particles will be dissolved by acidic water, resulting in the formation of secondary pores, and ultimately the formation of tight low permeability reservoirs^2–6^. In tight sandstone reservoirs, the wettability of formation water and natural gas to the rock surface is very different. The sandstone is wetted by aqueous phase, and the external fluid is easy to reside in the pore throat, while the oil and gas are wetted by non-aqueous phase. In addition, the content of various clay minerals in tight sandstone reservoirs is high, and clay minerals expand and disperse after encountering water, resulting in the reservoir being more vulnerable to water damage^7–11^. Therefore, tight sandstone gas reservoirs are generally characterized by low porosity (< 10%), low permeability (< 0.1 mD), small pore throat, serious heterogeneity, and micro-fracture development. They are extremely vulnerable to reservoir damage, and the damage is difficult to eliminate, which affects the correct evaluation and exploration and development of gas reservoirs.

In the process of oil and gas development, a large amount of drilling fluid will be used. Compared with other damage factors, the drilling fluid system is more likely to cause damage to tight sandstone reservoirs. The surface of the pore structure of the tight sandstone reservoir will adsorb some of the polymer treatment agents in the drilling fluid, blocking the pore throat structure, resulting in a decrease in reservoir permeability. At the same time, the interaction between drilling fluid and reservoir water, drilling fluid filtrate and formation water incompatible, contact to produce solid precipitation, into the reservoir micro cracks, causing reservoir damage^12–16^. Therefore, the formation of reservoir protection drilling fluid system^17,18^ is of great significance to reduce the damage to the reservoir during drilling. In addition, increasingly stringent environmental regulations require the use of environmentally friendly drilling fluid treatment agents and systems^19–21^. The tight sandstone gas reservoirs in the central Sichuan Basin are rich in reserves and play an extremely important role in oil and gas production. However, in the process of exploration and development, wellbore instability and reservoir damage occur frequently, which seriously restricts the efficient well construction in this area.

In this paper, the reservoir damage of tight sandstone gas reservoirs in Jinhua-Zhongtaishan block in central Sichuan is taken as the research object. The structural characteristics of the block are analyzed by means of characterization, and the reservoir damage factors are revealed. Based on the analysis of the potential damage and sensitivity damage mechanism of the reservoir, a new “Double protection” drilling fluid system with reservoir protection and environmental protection was constructed based on the traditional environmental protection drilling fluid system. The results of drilling fluid performance evaluation and reservoir protection performance evaluation show that the constructed “Double protection” drilling fluid system has stable rheological filtration before and after aging. At the same time, under this system, the core permeability damage rate and cuttings expansion rate are significantly reduced, and the cuttings recovery rate is also improved. The constructed “Double protection” drilling fluid achieves green and efficient exploration and development of tight sandstone gas reservoirs.

## Materials and methods

### Materials

Whole rock-mineral and clay mineral (Sampling in the determination target area), Series of salinity brine(homemade), Acid liquor (30 wt% HCl), Bentonite slurry, Sodium hydroxide (NaOH), Acrylate copolymer (JY-1B0, Polyurethane polymer (JHS-01), Allyl alcohol acrylic copolymer (YFKN), Potassium chloride (KCl), Lubricant (JHL-03), Barite (density 1.6–2.3 g/cm^3^), Methane (CH_4_), Surfactant.

### Methods

**Figure 1:**
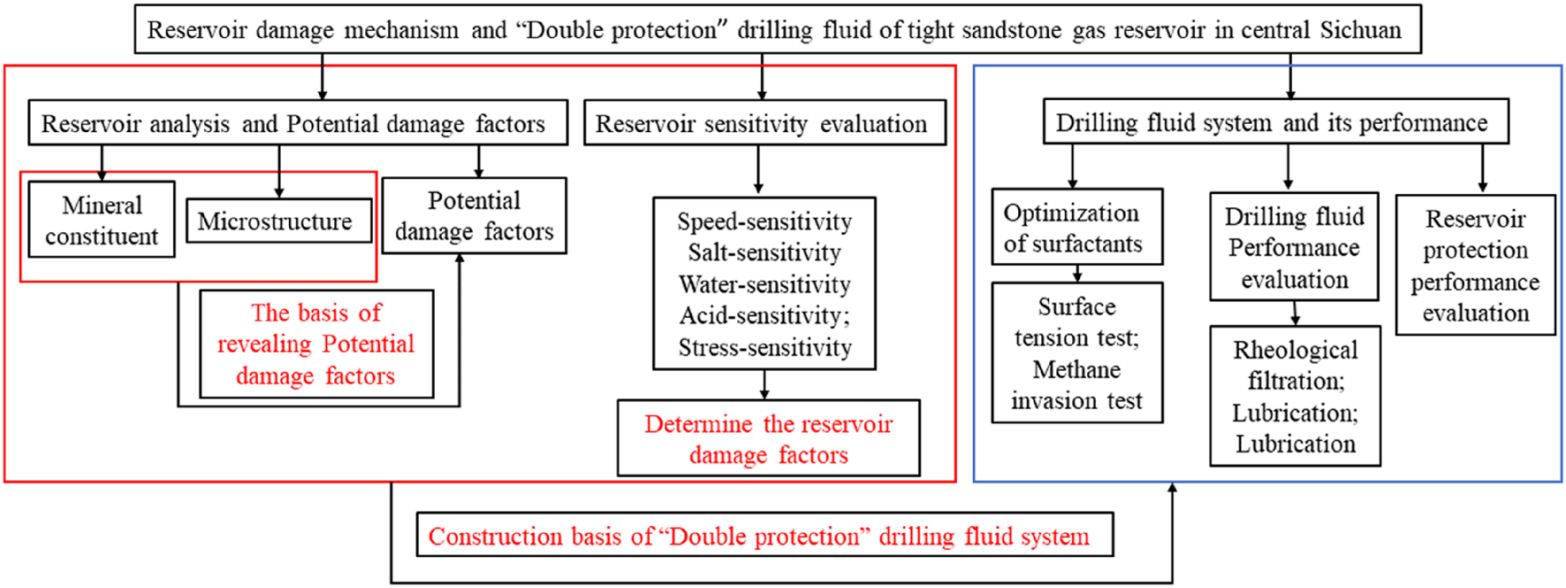
The working method roadmap.

In this paper, the working method is divided into three parts as shown in Fig. 1. Firstly, the reservoir rock structure of the block is analyzed. Based on the analysis results, the potential reservoir damage factors of the block are revealed. Further, through the sensitivity analysis of the reservoir in the block, the damage factors of the block are determined. Based on the above research results, a double-protection drilling fluid system was constructed by optimizing the surfactant, and the basic performance and reservoir protection performance of the drilling fluid were evaluated.

#### Synthesis of “Double protection” drilling fluid

A water-based drilling fluid was prepared by supramolecular polymerization using acrylate copolymer (JY-1B), polyurethane polymer (JHS-01) and allyl alcohol acrylic copolymer (YFKN) as the main raw materials. The basic formula is as follows: 4% bentonite slurry + 0.2% NaOH + 6% environment-friendly filtrate reducer JY-1B + 4% environment-friendly plugging agent JHS-01 + 0.3% environment-friendly inhibitor YFKN + 8% KCl + 2% lubricant JHL-03 + barite (density 1.6–2.3 g / cm^3^).

#### Analysis method of mineral structure

D/max-IIIA X-ray diffractometer was used to determine the relative content of whole rock minerals and clay minerals in the sampled cores of the target block. The microstructure characteristics of the cores sampled from the target block were analyzed by casting thin sections and scanning electron microscopy (FE-SEM, Hitachi SU-8200). The samples were tested by high pressure mercury intrusion, and the pore structure characteristics were analyzed.

#### Sandstone sensitivity evaluation method

Referring to SY/T5358-2010 “Reservoir sensitivity flow experiment evaluation method”, the velocity sensitivity, water-sensitivity, salt-sensitivity and acid-sensitivity of tight sandstone cores were tested at room temperature with simulated formation water as the flow medium. The specific experimental steps are as follows:

(1) Speed-sensitivity evaluation.

The completely saturated rock sample is loaded into the core holder to keep the flow direction of the liquid in the rock sample consistent with the flow direction of the gas when the gas permeability is measured, and to ensure that no air is left in the system during the whole experiment. Then the confining pressure is slowly adjusted to 2.0 MPa, and the confining pressure value is always greater than the core inlet pressure of 1.5–2.0 MPa during the detection process. The formation water flow rate is increased from 0.25 mL / min to 5.0 mL / min, and the permeability of rock samples at different flow rates is measured respectively. The permeability at the flow rate of 0.25 mL / min is used as the initial permeability to calculate the change rate of rock sample permeability with the increase of water flow rate.

(2) Water-sensitivity evaluation

The preparation process of the experiment is consistent with the preparation process of the speed-sensitivity evaluation. The initial liquid permeability of the rock sample was measured by the initial test fluid. With the intermediate test fluid displacement, the displacement speed is consistent with the initial flow rate. Displace 10 times to 15 times the pore volume of the rock, stop the displacement, keep the confining pressure and temperature unchanged, and make the intermediate test fluid fully react with the rock minerals for more than 12 h. The displacement pump flow rate is adjusted to the initial flow rate, and then the intermediate test fluid is displaced to determine the core permeability. The same method is used to replace the distilled water, and the permeability of the rock sample under the distilled water is measured.

(3) Salt-sensitivity evaluation

The preparation process of the experiment is consistent with the preparation process of the speed-sensitivity evaluation. The initial test fluid is used to determine the initial liquid permeability of the rock sample. After the initial liquid permeability of the rock sample is measured, the selected intermediate test flow breaks higher than the initial test fluid salinity are used to displace. The displacement speed is consistent with the initial flow rate, and the displacement is 10 times ~ 15 times the rock pseudo pore accumulation, stop the displacement, and keep the confining pressure and temperature unchanged. Make the brine fully react with rock minerals for more than 12 h; the flow rate of the displacement pump is adjusted to the initial flow rate, and then the same intermediate test fluid is displaced to determine the liquid permeability. The same force method is used to carry out the salt water displacement experiment under other salinity, and the highest salinity salt water displacement is carried out to determine the rock permeability under the corresponding salinity salt water.

(4) Acid-sensitivity evaluation

The preparation process of the experiment is consistent with the preparation process of the speed-sensitivity evaluation. The preparation process of the experiment is consistent with the preparation process of the speed-sensitivity evaluation. The liquid permeability of rock samples before acid treatment was measured by potassium chloride solution with the same salinity as formation water. Reversely inject 1.0 times the pore volume acid into the rock sample. Stop the displacement, close the inlet and outlet valves of the gripper, and make the reaction time between the rock sample and the acid 1 h. After the acid reacts with the rock sample, the potassium chloride solution with the same salinity as the formation water is positively displaced, and the liquid permeability of the rock sample after acid treatment is measured.

(5) Stress-sensitivity evaluation

The stress-sensitivity of reservoir rock is evaluated by variable confining pressure experiment. Taking the initial net stress as the starting point, the net stress is slowly increased according to the set net stress value. The net stress stops increasing when the net stress is added to the maximum net stress, and the net stress interval is set to 1.0 MPa, 1.5 MPa, 2.0 MPa, 2.5 MPa, 3.0 MPa, 3.5 MPa, 4.0 MPa, 4.5 MPa, 5.0 MPa, 5.5 MPa. Ensure that no less than 5 net stress points are set. Keep for more than 30 min at each set net stress point. After the net stress is added to the maximum net stress value. According to the net stress interval set by the experiment, the net stress is slowly reduced to the original net stress point. It should be maintained for 1 h at each set point of net stress.

#### “Double protection” drilling fluid performance test method

The surface tension of drilling fluid was measured by platinum ring method using JYW-200B automatic surface tension tester. The rheological properties of environmentally friendly water-based drilling fluid containing surfactant were determined by methane gas invasion experiment. The inhibition ability of the “Double protection” optimization system on the hydration dispersion of reservoir cuttings was evaluated by rolling dispersion experiment and linear expansion rate experiment. The reservoir protection performance of the optimized drilling fluid system was investigated by testing the gas permeability before and after the “Double protection” optimization system was polluted.

## Results and discussion

### Analysis results of potential damage factors of tight sandstone reservoir

#### Mineral composition analysis results

D/max-IIIA X-ray diffractometer was used to measure the relative content of whole rock minerals and clay minerals in the sampled cores of the target block, as shown in Table 1. From Table 1, it can be seen that the rock samples of tight sandstone reservoirs in SiChuan Basin are mainly quartz and plagioclase, and the content of clay minerals is low. The average quartz content is 38.33%, the average plagioclase content is 45.33%, and the clay content is distributed between 2 and 6%, with an average of 4%. In addition, it also contains a small amount of potassium feldspar and calcite and ankerite. The clay minerals are mainly composed of chlorite and illite/smectite mixed layer. The content of chlorite is between 35 and 75%, with an average of 59.67%. The content of illite-montmorillonite mixed layer is distributed between 10 and 54%, the average content is 27.33%, and the interlayer ratio is 20% -30%. The remaining clay minerals are illite.

**Table 1:** Mass fraction of whole rock minerals and the clay mineral mass percentage/%.

Quartz	Potash feldspar	Plagioclase	Calcite	Dolomite	Claymineral
36	12	41	2	3	6
35	8	49	1	3	4
44	7	46	1	–	2

Kaolinite	Chlorite	Illite	Illite-montmorillonitemixed-layer		Interlayingratio
0	35	11	54		30
0	69	13	18		20
0	75	15	10		20

#### Microstructure analysis

**Figure 2:**
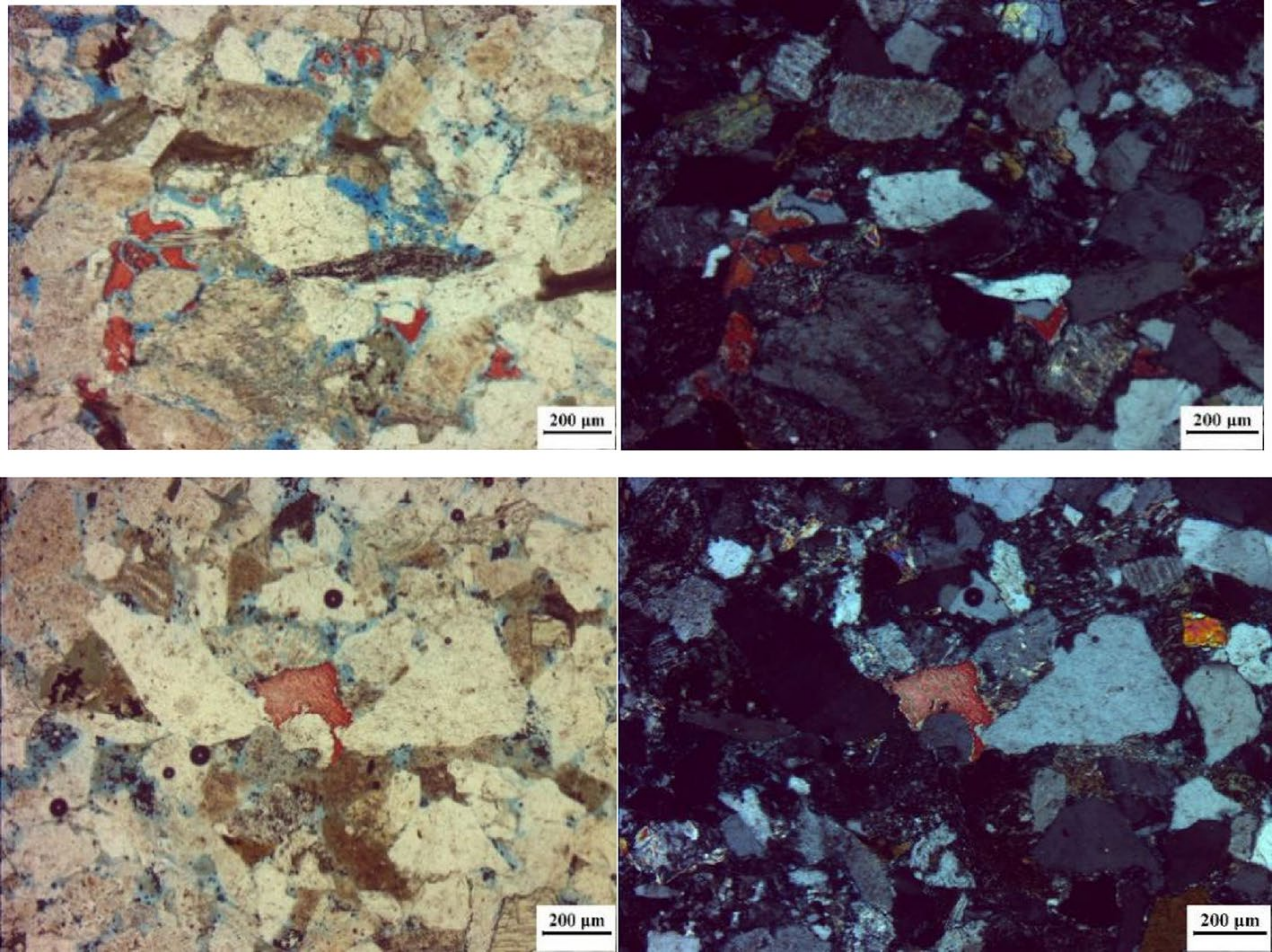
Casting thin sections of tight sandstone in central SiChuan basin.

**Figure 3:**
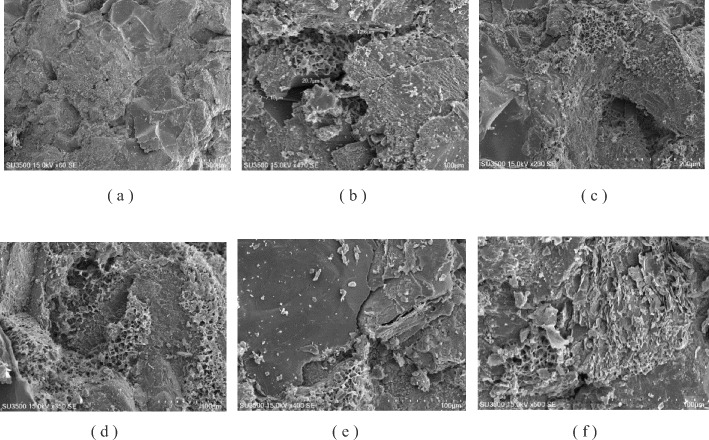
SEM images of tight sandstone in central SiChuan basin.

The microstructure characteristics of the core samples in the target block were analyzed by casting thin sections and scanning electron microscopy, respectively, as shown in Figs. 2 and 3. It can be seen from Fig. 2 that the rock samples in this block are medium sand-fine sandstone, with a small amount of calcite cementation, strong compaction, linear contact between particles, high content of plastic debris with plastic deformation characteristics, coexistence of primary pores and secondary pores, and low surface porosity, only 2.67%.

It can be seen from Fig. 3 (Fig. 3a: 60 times dense cementation, Fig. 3b: 470 times of dissolution pores, Fig. 3c: 230 times dissolution porelocal montmorilloniteization of particles, Fig. 3d: Partial montmorilloniteization of 350 times detrital particles, Fig. 3e: 400 times quartz crystal develops dissolution pores and fractures of 350 times detrital particles, Fig. 3f: Conversion of 500-fold feldspar particles to clay minerals) that the cementation of rock samples in this block is dense, and dissolution pores are seen. Feldspar particles are dissolved to montmorillonite, and clay minerals are montmorillonite.

**Figure 4:**
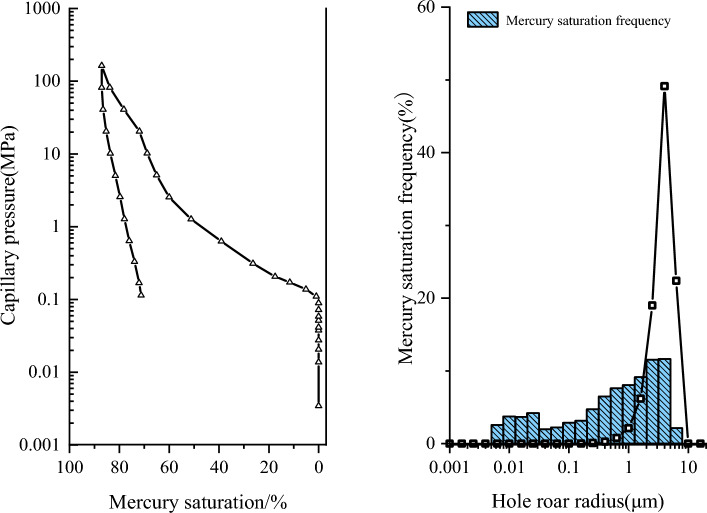
Mercury intrusion curve and pore throat distribution of tight sandstone in central SiChuan basin.

Further, the core of the target block was subjected to high-pressure mercury injection test to analyze the pore structure characteristics. The results are shown in Fig. 4. It can be seen from Fig. 4 that the displacement pressure of the sampled core in this block is 0.110 MPa, the maximum pore throat radius is 6.688 μm, the pressure is 1.207 MPa when the mercury saturation is 50%, and the corresponding median pore throat radius is 0.609 μm. The average pore throat radius of the core is 1.421 μm. The maximum mercury saturation of the core is 87.079%, and the mercury removal efficiency is 18.189%. The pore size distribution that contributes to permeability is between 1.60 μm and 6.30 μm.

#### Analysis of potential damage factors

Based on the above analysis results of rock fabric, the potential damage factors of tight sandstone gas reservoirs in Jinhua-Zhongtai Mountain Block in SiChuan Basin can be summarized as follows:

1. When the compositional maturity of rock is low, it is easy to produce stress-sensitivity under the action of pressure difference, which leads to the decrease of physical properties under the action of stress.
2. The pore throat size is small, and the distribution range is wide, the heterogeneity is strong, and the reservoir seepage ability is poor, which is easy to lead to the occurrence of “water lock” effect.
3. The content of clay in reservoir cores with poor physical properties is high. These clay minerals are distributed on the surface of particles or filled between pores, which are easy to react with the fluids invading the reservoir. Clay minerals such as illite are easy to fall off and migrate at high flow rates, thus blocking the throat and causing a “velocity-sensitive” effect.
4. Chlorite fills the pores, and there are a large number of intercrystalline micropores, resulting in a large capillary force, which is easy to cause liquid phase damage; in addition, chlorite, as an acid-sensitive mineral, is easy to fall off and block the pore throat under the action of acid etching, resulting in permeability damage.

### Sensitivity evaluation of tight sandstone reservoir

#### Speed-sensitivity evaluation

**Figure 5:**
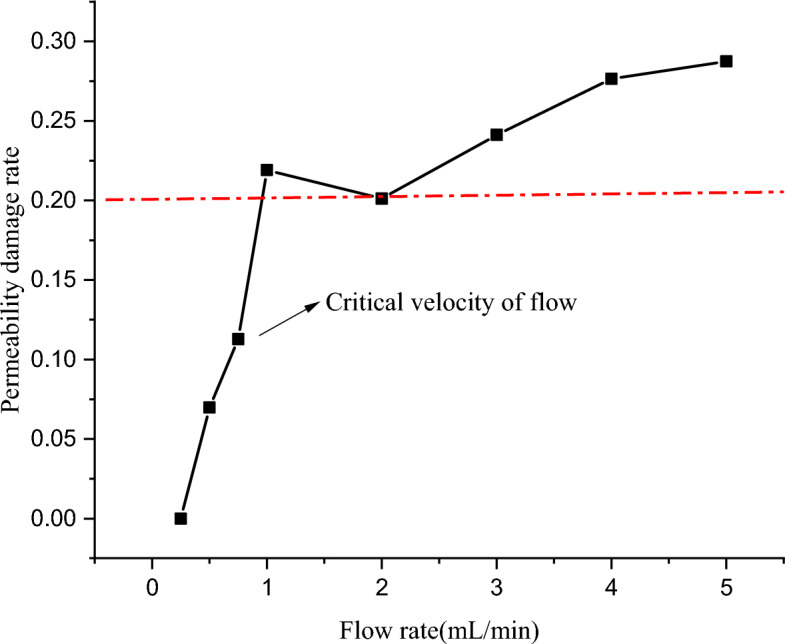
Results of flow velocity sensitivity experiment of tight sandstone in central SiChuan basin.

Referring to SY/T 5358–2010 “Reservoir Sensitivity Flow Experiment Evaluation Method”, the velocity sensitivity of tight sandstone cores was tested at room temperature with simulated formation water as the flow medium. The results are shown in Fig. 5. It can be seen from Fig. 5 that when the simulated formation water flow rate increases from 0.25 to 1.0 mL/min, the core permeability increases by 21.92%, and the flow rate sensitivity occurs. The critical flow rate is 0.75 mL/min, and the maximum permeability change rate is 27.65%. According to the damage evaluation standard, the speed-sensitive damage rate is within 30%, which is a weak speed-sensitive damage.

#### Water-sensitivity evaluation

**Figure 6:**
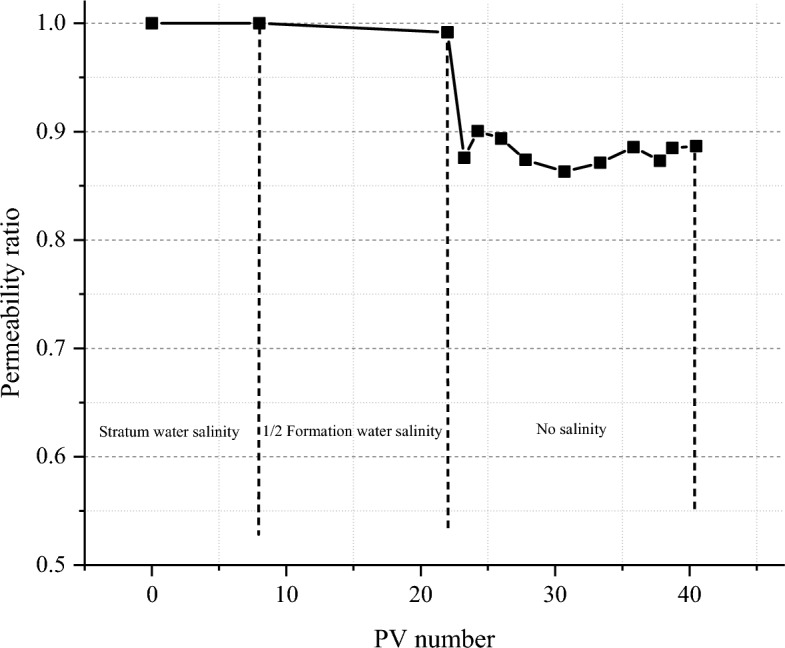
Results of water-sensitivity experiment of tight sandstone in central SiChuan basin.

The experimental results of water-sensitivity of tight sandstone cores in this block are shown in Fig. 6. After the intermediate fluid displacement, the permeability of the rock sample is basically unchanged, indicating that the low salinity has little damage to the core permeability. After flooding with distilled water, with the increase of PV number injected, the core permeability decreases, but the degree of decrease is not large, and the permeability damage rate is only 13.7%. According to the evaluation standard, the water-sensitivity damage degree of reservoir rock is weak, indicating that the sensitivity damage degree of low salinity is not high.

#### Salt-sensitivity evaluation

**Figure 7:**
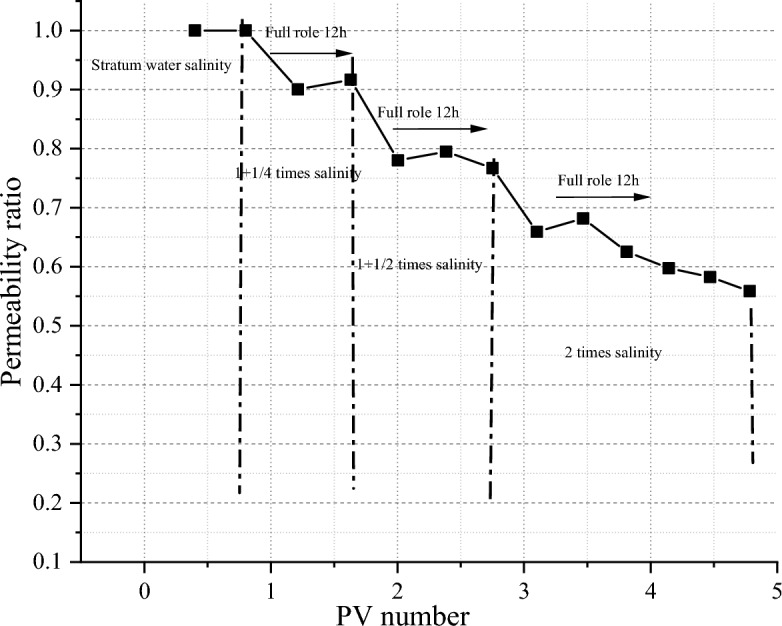
Results of salt-sensitivity experiment of tight sandstone in central SiChuan basin.

According to the results of water-sensitivity test, the maximum damage rate of water-sensitivity permeability is only 13.7%, so there is no need to carry out salinity reduction experiment. In view of the fact that the salinity of the test fluid is higher than that of the formation fluid, a series of salinity brine is selected for the sensitivity test of salinity increase. The results are shown in Fig. 7. The results show that the core is sensitive to high salinity fluid. After 12 h of full reaction under 1.25 times the salinity of formation water, the permeability damage rate of core fluid has reached 26.29%. Finally, when the salinity reaches 2 times the salinity of formation water, the core permeability damage rate has reached 56.33%. According to the sensitivity-damage evaluation index, the salt-sensitivity of reservoir rock is moderately strong. According to the standard of permeability reduction of 20%, it can be determined that the upper limit critical salinity is 1.125 times the salinity of formation water, that is, 9472.5 mg/L. The high salinity fluid compresses the thickness of the diffusion double layer of the clay particles, causing the particles to lose stability, fall off, block the pore throat, and cause the permeability to decrease.

#### Acid-sensitivity evaluation

**Figure 8:**
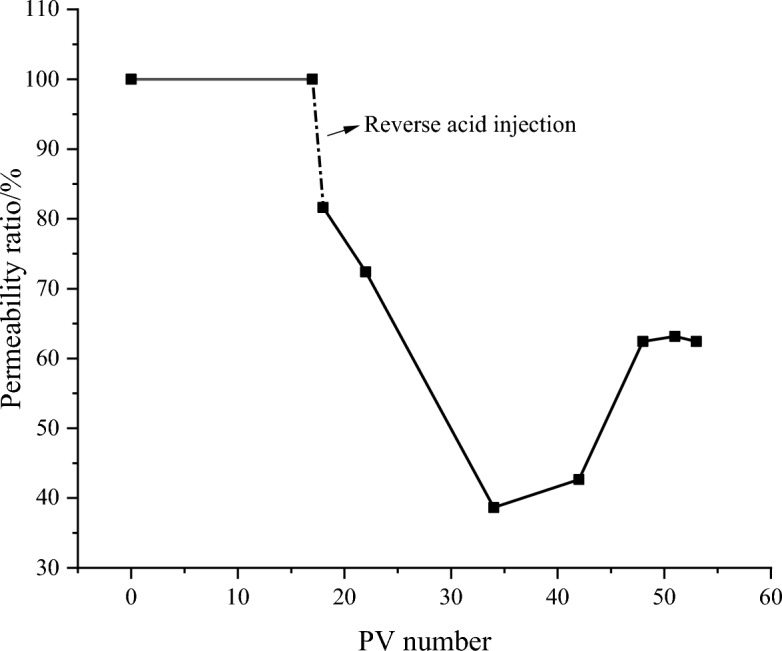
Results of acid-sensitivity experiment of tight sandstone in central SiChuan basin.

By reversely injecting 1 time the pore volume acid solution, and then closing the inlet and outlet, the rock sample is fully reacted with the acid solution for 1 h, and then the core is displaced in the positive direction to determine the liquid permeability of the rock sample after acid treatment. The results are shown in Fig. 8. The results show that after reverse acid injection, with the increase of cumulative injection multiples, the core permeability decreases first, and the damage degree reaches 63.16%. Then, when the PV number reaches 34 times, the permeability gradually recovers, and the final acid-sensitivity damage rate is 37.55%, which is moderately weak damage.

#### Stress-sensitivity evaluation

**Figure 9:**
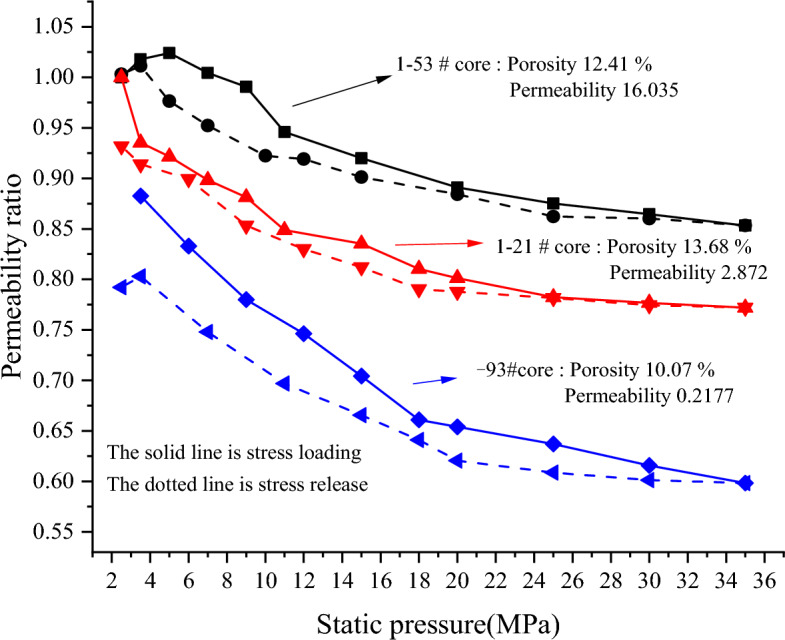
Results of stress-sensitivity experiment of tight sandstone in central SiChuan basin.

The stress-sensitivity of reservoir rock is evaluated by variable confining pressure experiment. The three cores evaluated represent three different physical properties of good, medium and poor, and the evaluation results are shown in Fig. 9. The core permeability will gradually decrease with the loading of stress. As the stress continues to increase, the degree of permeability damage gradually decreases. At the same time, the stress is released, and the permeability will be restored to a certain extent. The difference of physical properties significantly affects the degree of core stress-sensitivity. The permeability damage of the core with the best physical properties caused by stress is not more than 20%, and the permeability is basically restored after stress release, which is weak stress-sensitivity. The core with the worst physical properties is subjected to stress damage to a greater extent. As the stress is loaded to 35 MPa, the permeability damage reaches 40.167%, which is moderately weak stress-sensitivity.

According to the sensitivity evaluation results of tight sandstone gas reservoirs in Jinhua-Zhongtai Mountain Block, the main damage types of reservoirs in this block are summarized as follows:

1. The main damage type is salt sensitive damage, which is sensitive to high salinity fluid, and the critical salinity is 9472.5 mg/L.
2. The stress-sensitivity of reservoir cores has a strong correlation with physical properties. The stress-sensitivity damage of cores with good physical properties is weak, and the worse the physical properties are, the stronger the stress-sensitivity is. On the whole, the degree of stress sensitive damage belongs to weak sensitive-medium weak sensitive damage.

### Performance evaluation of “Double-protection” drilling fluid

The drilling fluid system used in the Jinhua-Zhongtaishan block in central SiChuan is an environmentally friendly desulfonated drilling fluid system. It is an environmentally friendly water-based drilling fluid based on the principle of supramolecular polymerization. The acrylic copolymer (JY-1B), polyurethane polymer (JHS-01), allyl alcohol acrylic copolymer (YFKN) as the main material, can quickly form a dense polymer film with good flexibility, high strength, and good adsorption self-healing in the drilling fluid, so that the drilling fluid has good plugging, inhibition and high temperature resistance. The basic formula is: 4% bentonite slurry + 0.2% NaOH + 6% environment-friendly filtrate reducer JY-1B + 4% environment-friendly plugging agent JHS-01 + 0.3% environment-friendly inhibitor YFKN + 8% KCl + 2% lubricant JHL-03 + barite (density 1.6–2.3 g/cm^3^).

**Figure 10:**
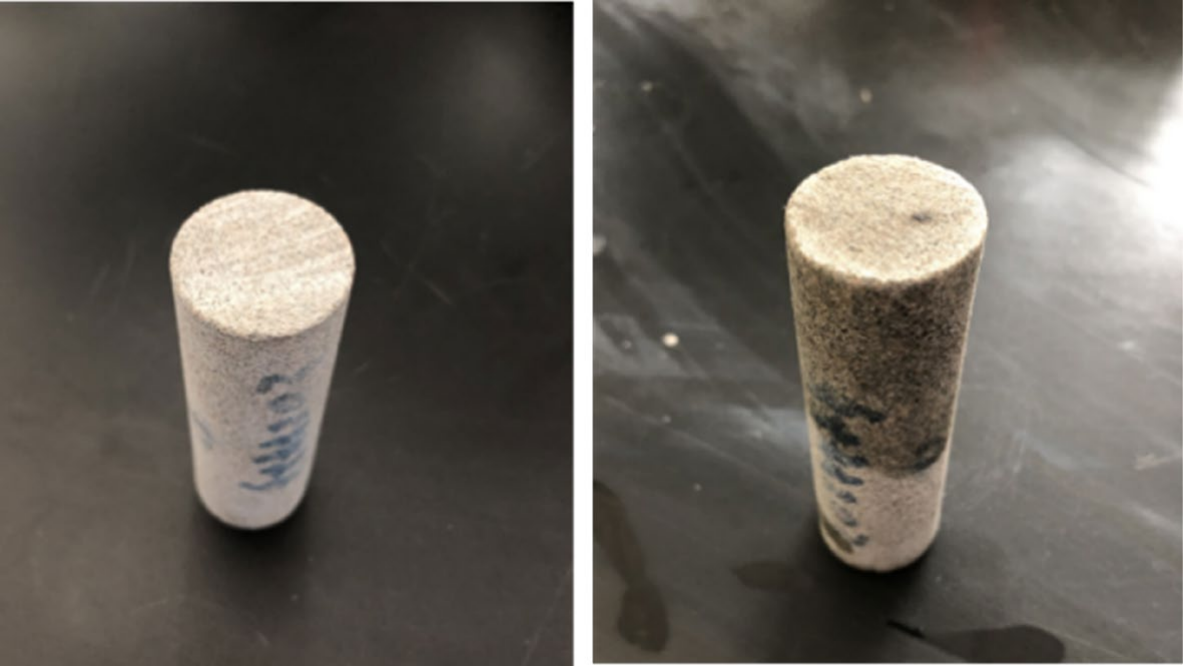
Core photos before and after contamination by field drilling fluid.

The field drilling fluid evaluation results show that the drilling fluid system has better rheological properties, inhibition performance, plugging and anti-collapse performance. However, the reservoir protection effect is poor, the permeability damage rate of the core is as high as 88.77%, and the liquid phase of the drilling fluid has obvious intrusion traces (Fig. 10). At the same time, gas invasion and degassing are difficult, the system performance deteriorates, and the reservoir damage is aggravated. Therefore, according to the geological characteristics and damage characteristics of tight sandstone in this block, the basic idea of optimizing the design of drilling fluid to protect the reservoir is to optimize the surfactant, reduce the surface tension, prevent the invasion of liquid phase, promote the flowback, and relieve the influence of methane gas invasion on the performance of the system.

#### Optimization of surfactants

(1) Surface tension test

**Figure 11:**
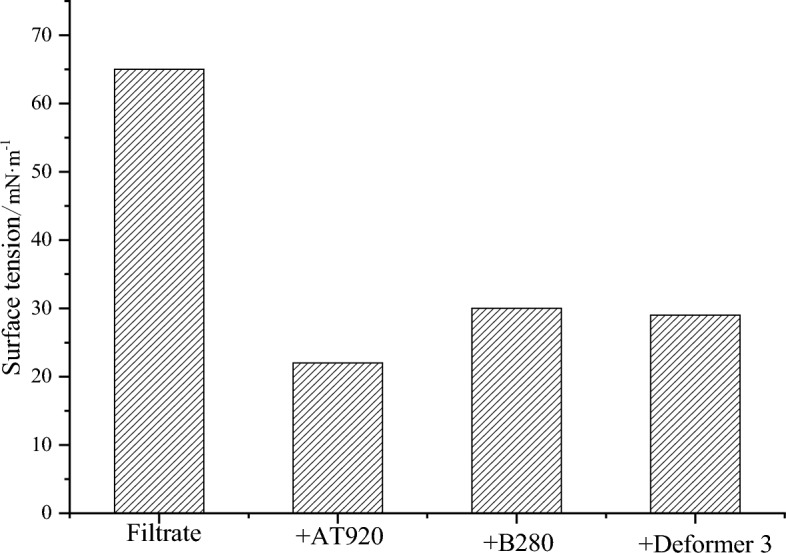
Surface tension test results.

The critical micelle concentration of silicone AT920 surfactant is 100 mg/L, and the concentration of B280 surfactant and Deformer surfactant is lower than 100 mg/L. Therefore, add 0.1% of each surfactant to the drilling fluid filtrate and test the surface tension. The results are shown in Fig. 11. It can be seen from Fig. 10 that each surfactant can reduce the surface tension of the filtrate. Among them, the silicone AT920 has the best surface tension reduction ability, which helps to enhance the anti-foaming ability of the drilling fluid, improve the degassing effect, and promote the filtrate flowback and strengthen the reservoir protection performance.

(2) Methane Gas Invasion Test

The AT920 was added to the environmentally friendly drilling fluid system to test its effect on the rheology of the system and the change of the rheological properties of the system during the gas invasion process. The results are shown in Table 2. It can be seen from Table 2 that AT920 has almost no effect on the rheology of the system, and the system can maintain stable rheology and density without foaming during the continuous gas injection process for 30 min, indicating that the preferred AT920 has a better anti-foaming effect.

**Table 2:** Effect of methane gas invasion on the rheological properties of efield drilling fluid containing surfactant.

System	Gas injection time/ min	AV (mPa·s)	PV (mPa·s)	YP (Pa)	Gel (Pa/Pa)	Ρ (g/cm3)
On-site environmentally friendly water-based drilling fluid + 0.1% AT920 (ρ = 1.85 g/cm^3^)	0	99	85	14	3.5/8	1.85
	5	100.5	86	14.5	4/8	1.85
	10	98	85	13	3/7.5	1.85
	20	100	86	14	4/8.5	1.85
	30	101	88	13	4/8	1.85

#### Basic performance evaluation of optimization system

Based on the preferred surfactant, a “Double-protection” water-based drilling fluid system with different densities suitable for the tight gas reservoir of this block was constructed through formula adjustment and optimization. The formula is as follows:

The density of 1.7 g/cm^3^ optimization system: 4% bentonite slurry + 0.2% NaOH + 0.2% xanthan gum XC + 0.3% zwitterionic polymer coating agent FA367 + 6% environmentally friendly fluid loss reducer JY-1B + 4% environmentally friendly plugging agent JHS-01 + 0.3% environmentally friendly inhibitor YFKN + 8% KCl + 2% lubricant JHL-03 + 0.1% AT920 + 1% viscosity reducer SF260 + barite (density 1.7 g/cm^3^).

The optimized system of density 2.3 g/cm^3^: 2% bentonite slurry + 0.2% NaOH + 0.2% zwitterionic polymer coating agent FA367 + 4% environmentally friendly filtrate reducer JY-1B + 4% environmentally friendly plugging agent JHS-01 + 0.2% environmentally friendly inhibitor YFKN + 8% KCl + 2% lubricant JHL-03 + 0.1% AT920 + 2% viscosity reducer SF260 + barite (density 2.3 g/cm^3^).

(1) Rheological filtration property.

The rheological filtration performance test results of the “Double-protection” optimized system before and after aging (90 °C/16 h) are shown in Table 3. From Table 3, it can be seen that the rheological filtration property of the optimized system is significantly improved compared with the original system. After formula adjustment and optimization, the rheological filtration performance of the system (density 1.7–2.3 g / cm3) meets the indicators : plastic viscosity ≤ 50 mPa·s, API water loss ≤ 5 mL, initial cut 2–5 Pa, final cut 3–10 Pa, dynamic shear force 5–12 Pa.

**Table 3:** Rheological and filtration performance results.

System	Condition	AV (mPa·s)	PV (mPa·s)	YP (Pa)	Gel (Pa/Pa)	FL_API_ (mL)	pH	Ρ (g/cm^3^)
The original system (*ρ* = 1.7 g/cm^3^)	Before aging	61	46	15	1/2	4	10	1.7
	Before aging	69.25	55.5	13.75	0.5/1	3.2	11	
The original system (*ρ* = 2.3 g/cm^3^)	Before aging	(over-range)	–	–	12/31	17.2	9	2.3
	Before aging	143.5	112	31.5	8/13.5	9.2	9	
“Double protection”optimization system (*ρ* = 1.7 g/cm^3^)	Before aging	33	24	9	2/5	3	10	1.7
	Before aging	35	25	10	3/5	2.4	10	
“Double protection”optimization system(*ρ* = 2.3 g/cm^3^)	Before aging	60	48	12	4/10	3.2	10	2.3
	Before aging	58	47	11	3.5/9	2.8	10	

(2) Lubricating property

The test results of the lubrication performance of the “Double protection”optimized system are shown in Table 4. It can be seen from Table 4, that the “Double protection” optimization system has good lubricity and is superior to the original system.

**Table 4:** Lubrication performance results.

System	Clear water reading	Sample readings	Correction factor	lubrication factor
The original system (ρ = 1.7 g/cm^3^)	32.27	10.6	1.0536	0.1117
The original system (ρ = 2.3 g/cm^3^)	32.23	12.9	1.0549	0.1361
“Double protection” optimization system (ρ = 1.7 g/cm^3^)	32.10	11.3	1.0592	0.1197
“Double protection” optimization system (ρ = 2.3 g/cm^3^)	35.33	10.5	0.9624	0.1010

(3) Inhibitory transmitter

**Figure 12:**
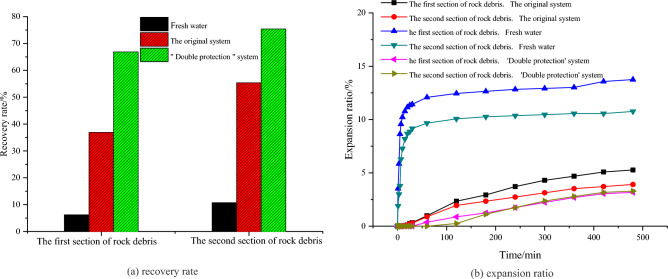
Inhibition evaluation results.

The cuttings of the first and second sections of the reservoir in this block were selected and processed into two specifications of 2–5 mm and over 100 mesh. The rolling dispersion experiment (90 °C/16 h) and linear expansion rate experiment were used to evaluate the inhibition ability of the “Double protection” optimization system to the hydration and dispersion of reservoir cuttings, and compared with the original system and clean water. The experimental results are shown in Fig. 12. It can be seen from Fig. 12. that the “Double protection” optimization system has excellent inhibition ability, which can improve the recovery rate of cuttings to more than 66% and reduce the expansion rate of cuttings to less than 3.2%.

#### Reservoir protection performance evaluation

**Figure 13:**
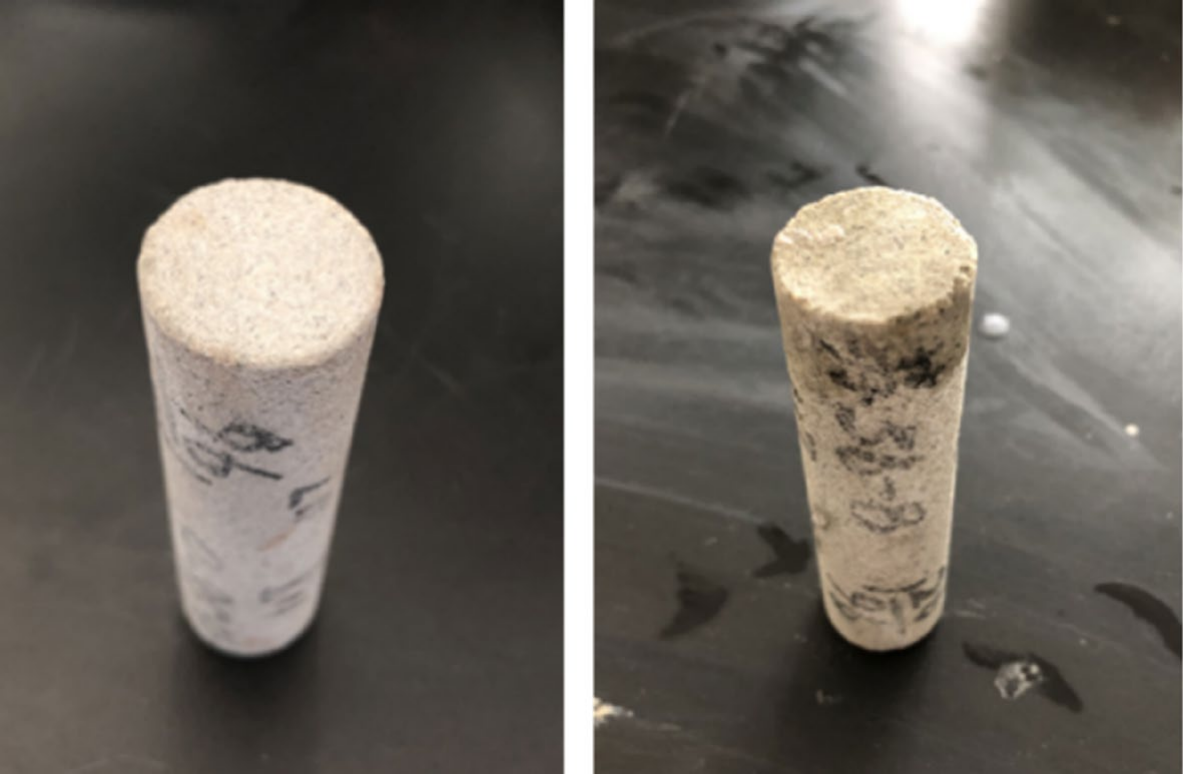
Core photos before and after contamination by optimized drilling fluid.

The tight sandstone core was selected to test the gas permeability before and after the pollution of the “Double protection” optimization system, and the reservoir protection performance of the optimized drilling fluid system was investigated. The experimental results are shown in Table 5. From Table 5, it can be seen that after the core is polluted by the “Double protection” optimization system, the permeability changes little, from 0.3087 mD to 0.2511 mD, and the damage rate is 18.66%, which is much lower than the damage rate of the original system. This shows that the optimized system has better reservoir protection performance. On the one hand, the system can form a good plugging layer and reduce the invasion depth (Fig. 13). On the other hand, the surfactant introduced in the system reduces the surface tension of the filtrate, which helps the filtrate to flow back and restore the permeability.

**Table 5:** Test results of core permeability damage rate.

Core number	Pollution system	Permeability before pollution/mD	Permeability after pollution/mD	Permeability damage rate/%	Dynamic filtration loss/mL
1–1	The original system (ρ = 1.7 g/cm^3^)	0.4205	0.0472	88.77	0
2–2	“Double protection”optimization system (ρ = 1.7 g/cm^3^)	0.3087	0.2511	18.66	0

## Conclusion

In this study, aiming at the problem of reservoir damage in the development of tight sandstone reservoirs, the geological characteristics and damage mechanism of Jinhua-Zhongtai mountain block in central Sichuan are studied. Based on the geological characteristics and damage mechanism of the block, a unique “Double protection” drilling fluid system is constructed. The results show that the reservoir in this block has poor seepage capacity and strong heterogeneity. The rock is mainly composed of quartz and plagioclase, with an average clay mineral content of 4%. The main damage type of the reservoir is salt-sensitive damage (Critical salinity of 9472.5 mg/L). The stress-sensitive damage is weak-moderate weak damage, and it also has a certain “Speed-sensitivity” and “Water-lock” effect. The constructed “Double protection” drilling fluid system has excellent comprehensive performance under the premise of environmental friendliness, and the permeability damage rate of tight sandstone core is reduced from 88.77% to 18.66%, and the reservoir protection effect is remarkable.

## Data Availability

All data generated or analysed during this study are included in this published article.
